# Fecal Bile Acids Profile of Crewmembers Consuming the Same Space Food in a Spacecraft Simulator

**DOI:** 10.3389/fphys.2021.593226

**Published:** 2021-10-01

**Authors:** Hai-Sheng Dong, Qi-Bing Shen, Hai-Yun Lan, Wei Zhao, Ping Cao, Pu Chen

**Affiliations:** ^1^State Key Lab of Space Medicine Fundamentals and Application, Key Laboratory of Space Nutrition and Food Engineering, China Astronaut Research and Training Center, Beijing, China; ^2^Innovation Center of Space Nutrition and Food Engineering, Shenzhen, China

**Keywords:** bile acids, prepackaged space food, fixed spacecraft simulator, metabolism, HPLC-MS-MS

## Abstract

**Introduction:** Recently, bile acids (BAs) are increasingly being considered as unique metabolic integrators and not just for the cholesterol metabolism and absorption of dietary lipids. Human BAs profiles are evolved to be individual under different environmental, dietary, and inherited factors. Variation of BAs for crewmembers from freshly prepared kitchen diets to wholly prepackaged industrial foods in a ground-based spacecraft simulator has not been clearly interpreted.

**Methods:** Three crewmembers were confined in a docked spacecraft and supplied with 7 days periodic wholly prepackaged industrial foods for 50 days. Fecal samples were collected before entry in the spacecraft simulator and after evacuation. Determination of 16 kinds of BAs was carried out by high-performance liquid chromatography tandem mass spectrometry method.

**Results:** Bile acids metabolism is sensitive to diet and environment transition from freshly prepared kitchen diets in the canteen to wholly prepackaged industrial foods in a ground-based spacecraft simulator, which is also specific to individuals. A significant positive relationship with a coefficient of 0.85 was found for primary BAs as chenodeoxycholic acid (CDCA) and cholic acid (CA), and a significantly negative relationship with a coefficient of −0.69 for secondary BAs as lithocholic acid (LCA) and deoxycholic acid (DCA).

**Discussion:** The profile of BA metabolism of individuals who share the same food in the same environment appears to be unique, suggesting that the inherent ability of different individuals to adapt to diet and environment varies. Since the transition from the free diet in open space to whole prepackaged space food diet in a space station simulator causes the variations of BAs pool in an individual manner, assessment of BA metabolic profiles provides a new perspective for personalized diet design, astronaut selection and training, and space flight diet acclimatization.

## Introduction

Bile acids (BAs) are synthesized in the liver with the gastrointestinal tract being the principal location where metabolism occurs. BAs are important endogenous substances in mammals with many important physiological functions, such as cholesterol balance, lipid absorption, carbohydrate metabolism, absorption of drugs and excretion of a toxic substance, immune regulation, and regulation of the composition of gut microbiota (Philippe et al., 2009; Piero et al., 2009; Genta et al., 2013). Recent studies have shown that the metabolic spectrum of BAs is an important manifestation of the function of the intestinal-hepatic axis. Changes to the metabolic spectrum of BAs are correlated with Alzheimer's disease, liver tumors, and colorectal cancer (Genta et al., 2013; Ji et al., 2019; MahmoudianDehkordi et al., 2019). Changes in the composition and quantity of BAs are of great importance to the study of nutritional metabolism since modification of BAs is directly mediated by the gut microbiota colonizing in the gastrointestinal tract, and fecal BA profiles are gaining recognition as a chemical indicator of many aspects of human health (Hoving et al., 2018; Molinaro et al., 2018). Other studies have shown that, even in the same environment, with the same diet, the gut microbiota of different individuals exhibit individual differences, including flexibility and recoverability (Faith et al., 2013; Turroni et al., 2017). Furthermore, it has been shown that variation in adaptability to isolated, confined, and extreme environments is related to individual differences (Bartone et al., 2018). A personalized diet has been recommended for the regulation of BAs homeostasis (Lozupone et al., 2012; Eggink et al., 2017; Wan and Jena, 2019). Gut microbiota are closely related to the metabolism of BAs, and diet can affect the composition and function of gut microbiota and the synthesis and secretion of BAs. The intestinal-hepatic circulation of BA depends on the reabsorption of BA by the end of the ileum and colon. Intestinal mucosal diseases can affect the reabsorption of BAs, the detection of fecal BAs can reflect intestinal mucosal function, and metabolic disorders of BAs play an important role in the development of intestinal inflammation. BAs not only participate in the physiological processes of cholesterol, lipid, and lipid-soluble molecules metabolism, but also play an important role as signaling molecules in regulating gut microbiota, intestinal mucosal immunity, and inflammation (Maslowski and Mackay, 2011). The intervention of metabolic homeostasis or metabolic diseases can be achieved by regulating the diet-gut microbiota-BA axis (Liu et al., 2015).

It has been reported that specific conditions during human-crewed space missions can cause changes in the gut microbiota (Saei and Barzegari, 2012). On the contrary, the degradation of BAs is directly regulated by the gut microbiota. However, it is unclear how composition profiles of BAs change due to variations in both the diet and space environment. In addition, due to the close correlation between BAs and individual inherent gut microbiota, those metabolic profiles of BAs in different crewmembers may also show specificity. Examination of the structure and concentration of BAs is of great significance in the diagnosis and treatment of metabolic diseases. Several studies have reported interactions between the gut microbiota, diet, and host health status (Murashita et al., 2018). The diverse metabolic function, postprandial monitoring, and regulation of BAs for crewmembers in human-crewed space flight mission conditions might be of importance but have been inadequately researched. For human-crewed space flight studies, the main drawbacks are the limited crewmembers in the spacecraft, which make extensive study based on the numerous individual subjects simultaneously not available (Lang et al., 2017). Meanwhile, repeated measurements of individuals during longitudinal research, especially for samples of the twins also serve as an efficient way to follow (Garrett-Bakelman et al., 2019). The structure of fecal BAs is closely related to host diets, environments, and inherent physiological status. Unlike routine diets on earth, the food that astronauts consume in space is mostly prepackaged food prepared on the ground and transported to astronauts through cargo spaceships. We assume that the dietary transitions from freshly prepared food on the ground to whole prepackaged food in space lead to variations in the metabolism profile of BA. Here, we carried out the project in a fixed ground-based space station simulator where the three crewmembers were confined and consumed the same prepackaged food for 50 days. During the project, fecal samples were collected at scheduled intervals according to the recipe cycle. A total of 16 kinds of BAs, including primary, secondary, and conjugated forms were analyzed by high-performance liquid chromatography tandem mass spectrometry. In this study, the variation in composition profiles of BAs of three crewmembers was analyzed when given the same prepackaged space food diet in a fixed ground-based space station simulator.

## Materials and Methods

### Subjects

Major information of the three crewmembers (C01, C02, and C03) in the research and proportion of energy supply of the scheduled periodic diet served as shown in Table S1. The crewmember could eat freely under the guidance of the Chinese resident diet pagoda outside the simulator (OS), and the whole prepackaged food recipe (for details see Supplementary Table S2) was provided inside the simulator (IS). The fecal samples were gathered at various time intervals throughout the entire experiment according to the schedule, as shown in Supplementary Figure S1. In order to obtain the fecal samples from crewmembers, a disposable stool collection bag was provided for daily stool collection and processing. The crewmembers were asked to collect the fecal samples at different time points before entry (S1 refers to the sampling time point 72 days before entering), IS (sequenced symbol S2 to S9 refers to the sampling time points on days 2, 9, 16, 23, 30, 37, 44, and 50 IS), and after exiting the simulator (S10, S11, and S12 refer to sampling time points on days 10, 15, and 20 after evacuation). In particular, for each sampling time point IS, the collected samples were transferred to a −80°C freezer for further analysis by a mobile −20°C freezer IS. This study was reviewed and approved by the Ethics Committee of the China Astronaut Research and Training Center (Permission No. ACC18SP01). All the volunteers gave written informed consent prior to their inclusion in the study. All the methods were performed in accordance with the relevant guidelines and regulations.

### Equipment and Materials

Determination of the fecal BAs was carried out according to the liquid chromatography tandem–mass spectrometry method described by Hagio et al. with minor modifications (Masahito et al., 2009). Briefly, about 10 mg fecal samples were precisely weighed by microanalytical balance (METTLER TOLEDO XPR26DR/AC, Polaris Parkway Columbus, USA); 1 ml methanol was added to precipitate protein. After vortex oscillation by a blending apparatus (Qilinbeier QL866, Qilinbeier, Haimen, Jiangsu provice, China) for 1 min and centrifuged at 4°Cand 12,000 g by 21CR high-speed refrigerated centrifuge (Thermo Fisher Scientific Co., Ltd., Waltham, MA, USA) for 10 min, the supernatant was diluted for one time and then analyzed by Waters ultra performance liquid chromatography liquid phase analyzer (Waters Acquity, Milford, MA, USA) coupled with AB 4000 triple quadrupole mass spectrometer (AB 4000, Framingham, MA, USA). The chromatographic column was ACQUITY UPLC®BEH, Milford, MA, USA C18 column (2.1 × 100 mm, 1.7 μm, Waters), with the sampling volume of 5 μl, column temperature of 40°C, mobile phase A of 0.01% formic acid water, mobile phase B of acetonitrile. Gradient elution conditions included 25% B for 0–4 min, 25–30% B for 4–9 min, 30–36% B for 9–14 min, 36–38% B for 14–18 min, 38–50% B for 18–24 min, 50–75% B for 24–32 min, 75–100% B for 32–35 min, 100–25% B for 35–38 min, with the flow rate of 0.25 ml/min. Mass spectrometric conditions were electrospray ionization source and negative ion ionization mode. The temperature of the ion source was 500°C, the voltage of the ion source was −4,500 V, the collision gas was 6 psi, the curtain gas was 30 psi, and the atomization gas and the auxiliary gas were 50 psi. Multiple reaction monitoring was used for scanning. A total of 16 kinds of reference substances of BAs (purity ≥ 98%), methanol, acetonitrile, and formic acid were all chromatographic pure grade (Merck Company, New Jersey, NJ, USA). HPLC water was super pure water made by Milli-Q Integral pure water system (Millipore Company, California, CA, USA). A list of the 16 kinds of BAs investigated in this study was shown in Supplementary Table S3

### Statistical Analysis

The BAs profile analysis and visualization were carried out by an online statistical analysis platform, namely, Calypso Version 8.84, Berghofer, Turrbal, Australia (Zakrzewski et al., 2016). The distance measure method for principal coordinates analysis (PCoA) of BAs was the Bray-Curtis, and the statistical method was repeated measurement ANOVA with a *p-*value cutoff of 0.05. The distance measure method for correlation analysis was Pearson's *r*.

## Results

**Figure 1:**
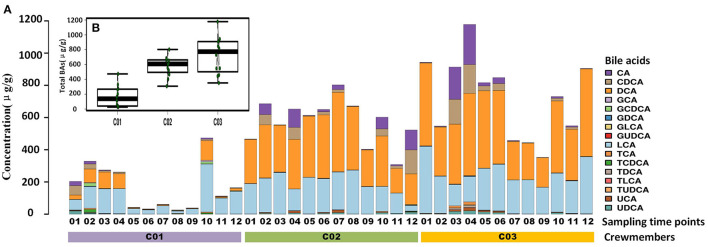
Profiling of fecal bile acids (BAs) of the three crewmembers. **(A)** Longitude variation of fecal BAs of the three crewmembers (C01, C02, and C03 refers to the three crewmembers, sequenced numbers 01–12 refers to different sampling time pionts). **(B)** Comparison of total BAs from fecal samples from the three crewmembers.

**Figure 2:**
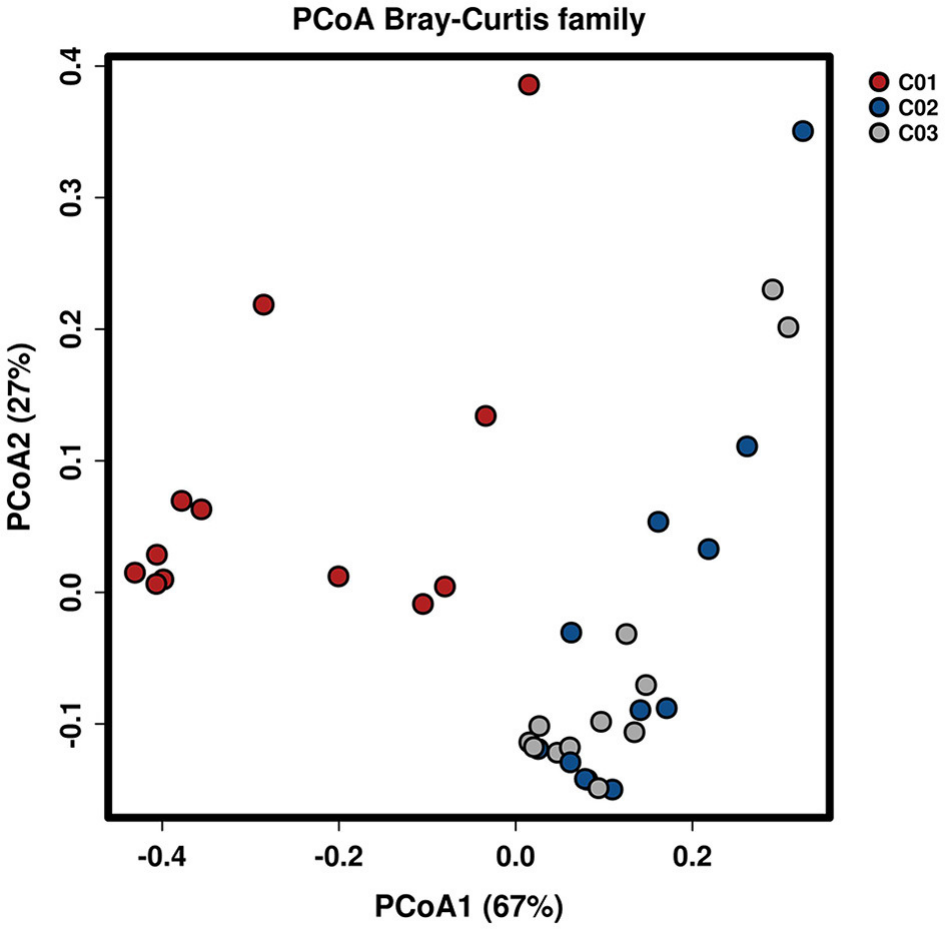
Principal coordinates analysis of BAs in the fecal samples of the three crewmembers (C01, C02, and C03 refers to the three crewmembers).

**Figure 3:**
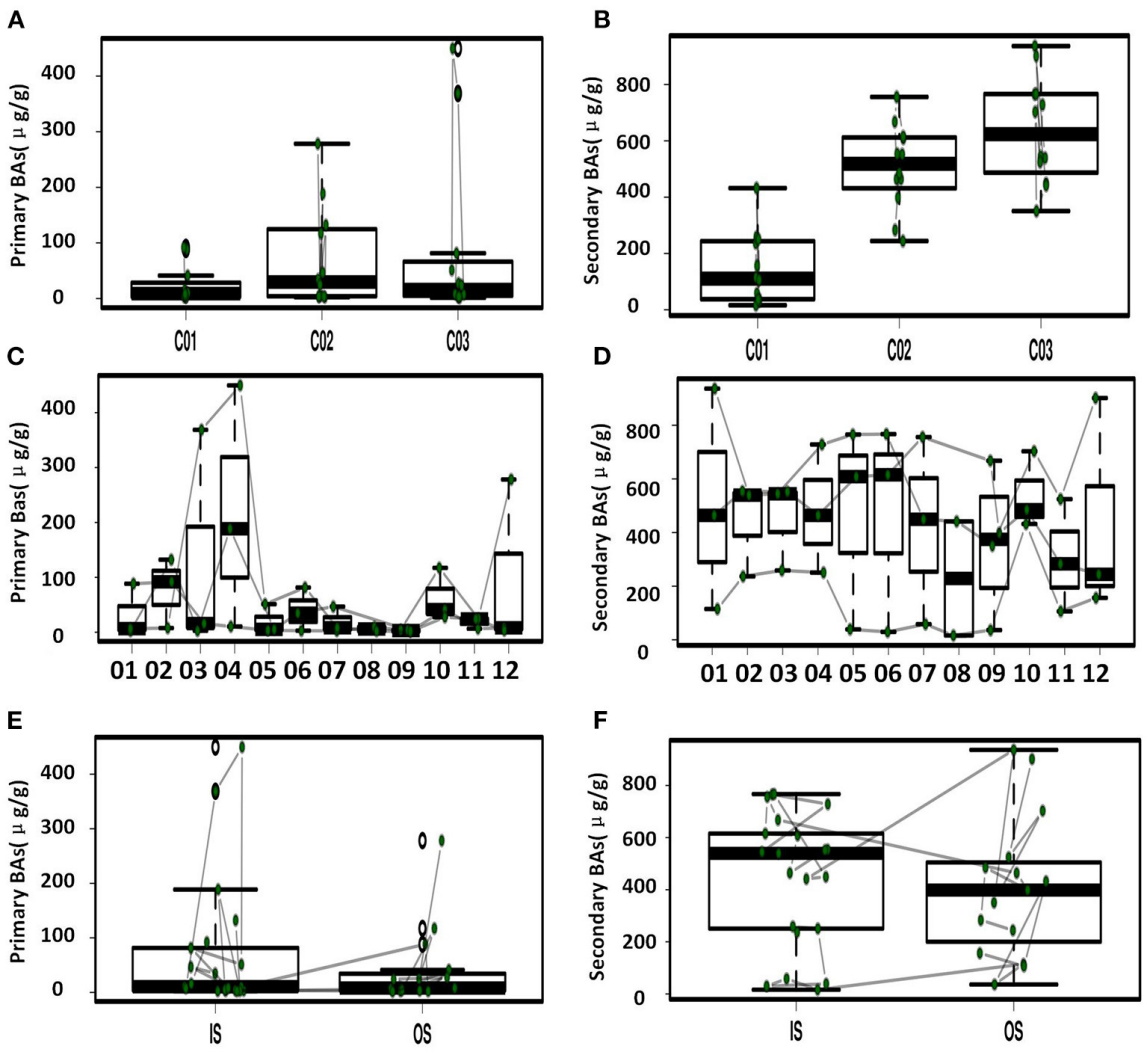
Primary and secondary BAs in feces with significant difference. **(A)** Comparison of primary BAs for the three crewmembers. **(B)** Comparison of secondary BAs for the three crewmembers. **(C)** Temporal variation of primary BAs at the different sampling time points. **(D)** Temporal variation of secondary BAs at the different sampling time points. **(E)** Comparison of primary BAs in the simulator (IS) and out of the simulator (OS). **(F)** Comparison of secondary BAs IS and OS.

Longitude variation profiling of the fecal BAs of the three crewmembers during the experiment was shown in Figure 1 and Supplementary Figure S2. First, we found that the fecal BAs profiles of the three crewmembers were different (see Figure 1A) when living in a space station simulator and consuming the same food. The quantity of total BAs in feces from each crewmember in the descending order (see Figure 1B) was crewmember 03 > crewmember 02 > crewmember 01. The analysis using repeated measurements ANOVA demonstrated significant differences (*p* < 0.05) between crewmembers, indicating that the synthesis and metabolism intensity of BAs in the crewmembers were different. Structure profiling of BAs pool for each crewmember was shown in Supplementary Figure S3. The BAs profile of crewmember 01 was quite different from that of crewmembers 02 and 03. The order of dominant BAs abundance for crewmembers 01 were: lithocholic acid (LCA) > deoxycholic acid (DCA) > chenodeoxycholic acid (CDCA) > cholic acid (CA). The composition of the pool of BAs of the crewmembers 02 and 03 was similar, with dominant order of abundance of BAs being as follows: DCA > LCA > CA > CDCA. The proportion of non-dominant BAs, such as glycodeoxycholic acid (GDCA), glycochenodeoxycholic acid (GCDCA), taurodeoxycholic acid (TDCA), glycocholic acid, taurochenodeoxycholic acid (TCDCA), taurocholic acid (TCA), and taurolithocholic acid was different in the three crewmembers, indicating that there were individual characteristics in the composition of pools of BAs. PCoA profiling of metabolites of BAs in the fecal samples of the three crewmembers was shown in Figure 2. Crewmembers 02 and 03 had consistency in the structure and level of BAs, while crewmember 01 was unique in the structure and level of BAs. BAs profiles with significant differences between crewmembers were shown in Figures 3A,B. As shown in Supplementary Figure S4, the levels of conjugated BAs (TCA, TCDCA, and GCDCA) of crewmember 01 were significantly higher than those of crewmembers 02 and 03 (*p* < 0.01), while the levels of total BAs, LCA, and DCA of crewmember 01 were significantly lower than those of crewmembers 02 and 03 (*p* < 0.01).

Temporal variation dynamics of the fecal BAs during the experiments was shown in Figures 3C,D and Supplementary Figure S5. After the transition from free eating OS to the prepackaged food diet IS environment, the total levels of BAs in feces first increased and then decreased to the original level stepwise before entering and after exiting the simulator. The fecal primary BAs exhibited the same trend (Supplementary Figure S5), while the secondary BAs varied in the opposite direction, indicating that the metabolic processes related to BAs increased during the period IS, with an increase in the conversion of primary BAs to secondary BAs. The time point at which a change in GCDCA and TCDCA levels was observed was close to the sampling point when the dietary pattern changed, namely, the first sampling (S2) after eating prepackaged food upon entering the simulator and the first sampling (S10) of free eating freshly prepared food after leaving the simulator. This indicates that the metabolic spectrum of BAs in the crewmembers was significantly affected by the change in the dietary pattern. We can conclude from Figure 1 and Supplementary Figure S5 that the size and composition of the pool of BAs for the three crewmembers were altered by the intake of prepackaged food in the fixed spacecraft simulator. The phenomenon was also verified by the temporal variation dynamics of BAs during the experiments shown in Figures 3C,D, since the time points at which the levels of BAs changed were mostly close to the sampling points when the environments and diets changed. This pattern common to all the three crewmembers was that the total quantity of BAs in their feces changed significantly when IS, which was recovered by sampling time point S12 after the experiment to the same level that existed before entering the simulator, indicating that the metabolism of BA is sensitive to the diet and environment, which is also specific to the individuals. Higher levels of primary BAs such as CA and CDCA were the unique characteristics for crewmember 02. Taking the influence of environment, diet, individual variation, and time duration together, we contrasted the fecal BAs levels of crewmembers during the period IS and that OS. The result was shown in Figures 3E,F and Supplementary Figure S6. The total BAs, CA, CDCA, DCA, and LCA levels IS were significantly higher than those OS.

As shown in Table 1, significant correlations were observed among various BAs, indicating regulation of dynamic balance and adaptability of metabolic pools of BA to the transition of diet and environment. A significant positive relationship (*p* < 0.05) with a coefficient of 0.85 was found for primary BAs, CDCA, and CA. The quantity of the two most dominant lipophilic secondary BAs (LCA and DCA) was significantly negatively correlated with a correlation coefficient of −0.69 (*p* < 0.05). There was a significant positive correlation between glycine-conjugated BA and taurine-conjugated BA (*p* < 0.01), with a correlation coefficient of 0.79.

**Table 1:** Correlation coefficien matrix of dominant bile acids (BAs) and their conjugated form.

**BAs**	**Gly-BA**	**Tau-BA**	**CDCA**	**CA**	**LCA**	**DCA**
Gly_BA	NA					
Tau_BA	0.79^*^	NA				
CDCA	0.13	0.26	NA			
CA	−0.07	0.15	0.85^*^	NA		
LCA	0.00	0.12	−0.49^*^	−0.57^*^	NA	
DCA	0.01	−0.01	−0.27	−0.10	−0.69^*^	NA

*NA, Not applicable*.

* *Significant correlation (p < 0.05)*.

## Discussion

In hepatocytes, BAs synthesized directly from cholesterol are termed as primary BAs, including CA and CDCA. Conjugated BAs are discharged into the intestinal tract to form free BAs by debonding by bacteria such as *Bacteroides, Clostridium, Lactobacillus, Bifidobacterium*, and *Listeria*, and then dehydroxylated by *Clostridium* and *Eubacterium* to form secondary BAs such as LCA and DCA (Mullish et al., 2018; Song et al., 2019). Human BAs pool includes primary BAs such as CA and CDCA and secondary BAs such as DCA and LCA. BAs can also become conjugated with glycine and taurine. The conjugated BAs are water-soluble amphoteric molecules that are easily soluble in the acidic environment of the intestine with a strong emulsification action, able to emulsify lipids in the intestinal cavity into particles that increase the contact area between lipid and lipase in the digestive juices in order to promote digestion and the absorption of lipids and the fat-soluble vitamins (Philippe et al., 2009). After dietary intake, these are secreted into the intestine, where they perform an important role in regulating the metabolism of carbohydrates, lipids, etc., *in vivo* (Molinaro et al., 2018). The regulation of BA *in vivo* is a very complex process, which requires the joint action of the liver, intestinal tract, and gut microbiota (Molinaro et al., 2018). Reabsorption, excretion, and synthesis of BAs during enterohepatic circulation allow the maintenance of a dynamic BAs pool balance (Philippe et al., 2009). In this study, the effect of prepackaged food intake on the BAs profile of feces of crewmembers was investigated for the first time in a fixed spacecraft simulator environment.

The profile of metabolism of BAs of different individuals sharing the same food in the same environment was unique, suggesting that the inherent ability of different individuals to adapt to the diet and the environment was different. The time point where the maximum total BAs occurred was different for the three crewmembers. The peak total BA level in crewmember 01 occurred on the second day IS, but on the 37th and 16th day and for crewmembers 02 and 0,3 respectively, suggesting that during the 50-day experiment, the metabolic spectrum of BAs for the crewmembers was flexible and exhibited intrinsic attributes when placed under the same dietary intervention conditions.

Temporal variation dynamics of BAs during the experiments show that the quantity of BAs and structure respond quickly to the diet and environment transitions and are recoverable stepwise once the original environment is reestablished. The significant contemporary variation of both the primary BAs (CA and CDCA) and the secondary BAs (DCA and LCA), indicates that the synthesis and metabolism of BAs were affected by the specific diet and environment. Both the host and gut microbiota regulate the size of BAs pools. Through hydrolysis of BAs brine and 7α-dehydroxylation, gut microbiota transform primary BAs synthesized by the host liver into secondary BAs such as DCA and LCA (Fiorucci and Distrutti, 2015). Of these, higher concentrations of the secondary BAs as LCA and DCA or primary BAs as CA and CDCA might be closely related to the abundance and activity of 7α-dehydroxylase secreted by the gut microbiota (Sheng et al., 2017; Song et al., 2019). Intestinal bacteria such as *Clostridium scindens* and *Lostridium sordellii* can secrete BAs 7α-dehydroxylase, and secondary BAs such as LCA and DCA formed by enzymatic hydrolysis of primary BAs can assist antibiotics inhibition of the growth of *Clostridium difficile* that are known to cause intestinal inflammation (Genta et al., 2013; Studer et al., 2016; Sheng et al., 2017). The increase of primary BAs levels during the period of the simulator stay of crewmembers was possibly due to changes in the transformations and metabolism of primary BAs associated with variations in the gut microbiota that were caused by the change in diet (Turroni et al., 2017; Jena et al., 2018). An increase of the level of secondary BAs IS, indicates the decrease of reabsorption process of BAs or the increase of transformation of primary BAs (Fiorucci and Distrutti, 2015; Molinaro et al., 2018). Conjugated BAs have excellent solubility properties, which play an important role in lipid digestion by acting as tensioactives, molecules that have both hydrophilic and lipophilic chemical groups (Shang et al., 2017; Molinaro et al., 2018). As shown in Supplementary Figure S5, an increase in the fecal GDCA and TDCA levels during the transition of diet might be related to a decrease in the process of ileal reabsorption, since most of the BAs are principally reabsorbed in the ileum (Zhu et al., 2019; Berg et al., 2020).

There were significant correlations among various BAs, indicating the dynamic balance regulation and adaptability of BA metabolic pools. BAs are reabsorbed at the end of the ileum by active transport, resulting in their accumulation as a dynamic pool in the body that circulate between the liver and the intestine, with ~95% reabsorbed in each cycle, while ~5% of these are excreted along with stool (Fiorucci and Distrutti, 2015). There is a close correlation and dynamic equilibrium in the concentration changes of the compounds of BAs.

Gut microbiota that secrete bile salt hydrolase (BSH) and 7α-dehydroxylase are responsible for the formation of secondary BAs through the metabolism of primary BAs. The disruption of homeostasis of BA plays a key role in intestinal inflammation (Fu et al., 2019). On the contrary, if the gut bacteria containing BSH and 7α-dehydroxylase are too numerous, the formation of a large number of cytotoxic secondary BAs, especially DCA, will cause DNA damage by the production of reactive oxygen species, the promotion of cell proliferation, a reduction in apoptosis and differentiation, and also the promotion of colorectal cancer (Wang et al., 2018). Changes in the levels of LCA and DCA in feces indicate that the composition of the gut microbiota might have changed accordingly. BAs, especially, taurine and glycine-conjugated BAs, can perform both proinflammatory and anti-inflammatory roles by activating the G-protein-coupled receptor on the surface of both the cell types, respectively (Pols et al., 2010). The positive correlation between glycine-conjugated and taurine-conjugated BAs suggests a balance of regulation of inflammation (Pols et al., 2010; Murakami et al., 2015). Synthesis and dysbiosis have previously been implicated in inducing systemic inflammation and reducing neuroplasticity in specific diets such as the western diet that could be related to cognitive dysfunction (Jena et al., 2018).

The purpose of this study was to investigate changes in the BA metabolism profile under the combined effects of the diet and environmental changes in simulated human-crewed space missions. We found that even when in the same environment for an extended period and consuming the same diet, metabolism of BAs still has individual attributes. Multiple rounds of such trials should be further carried to figure the common variations and mechanisms. Since the transition from free diet in open space to whole prepackaged space food diet in a space station simulator alters the BAs pool on an individual basis, assessment of BA metabolic profiles provides a new perspective for the personalized diet design, astronaut selection and training, and space flight diet acclimatization.

## Data Availability Statement

The original contributions presented in the study are included in the article/Supplementary Material, further inquiries can be directed to the corresponding author/s.

## Ethics Statement

The studies involving human participants were reviewed and approved by the Ethics Committee of China Astronaut Research and Training Center. The patients/participants provided their written informed consent to participate in this study. Written informed consent was obtained from the individuals for the publication of any potentially identifiable images or data included in this article.

## Author Contributions

H-SD and Q-BS made an equal contribution to the research. H-SD, WZ, and PCa provided the concept and design of the research. H-SD and Q-BS performed the experiments, analyzed the data, and prepared the figures. PCh got involved in drafting the manuscript. All authors contributed to the article and approved the submitted version.

## Funding

This project was funded by the Key Laboratory of Space Medicine Fundamentals and Applications (SMFA16B03), the Space Gut Research Project (BZZ20J001), and the Shenzhen Science and Technology Plan Project (JCYJ20170818100846805, JCYJ20180305163647462, JCYJ20180507182854651).

## Conflict of Interest

The authors declare that the research was conducted in the absence of any commercial or financial relationships that could be construed as a potential conflict of interest.

## Publisher's Note

All claims expressed in this article are solely those of the authors and do not necessarily represent those of their affiliated organizations, or those of the publisher, the editors and the reviewers. Any product that may be evaluated in this article, or claim that may be made by its manufacturer, is not guaranteed or endorsed by the publisher.
