# Supplementary figures and images for: Fecal Bile Acids Profile of Crewmembers Consuming the Same Space Food in a Spacecraft Simulator
Source: Front Physiol. 2021 Oct 1;12:593226. doi: 10.3389/fphys.2021.593226 (PMC8517451; doi:10.3389/fphys.2021.593226)

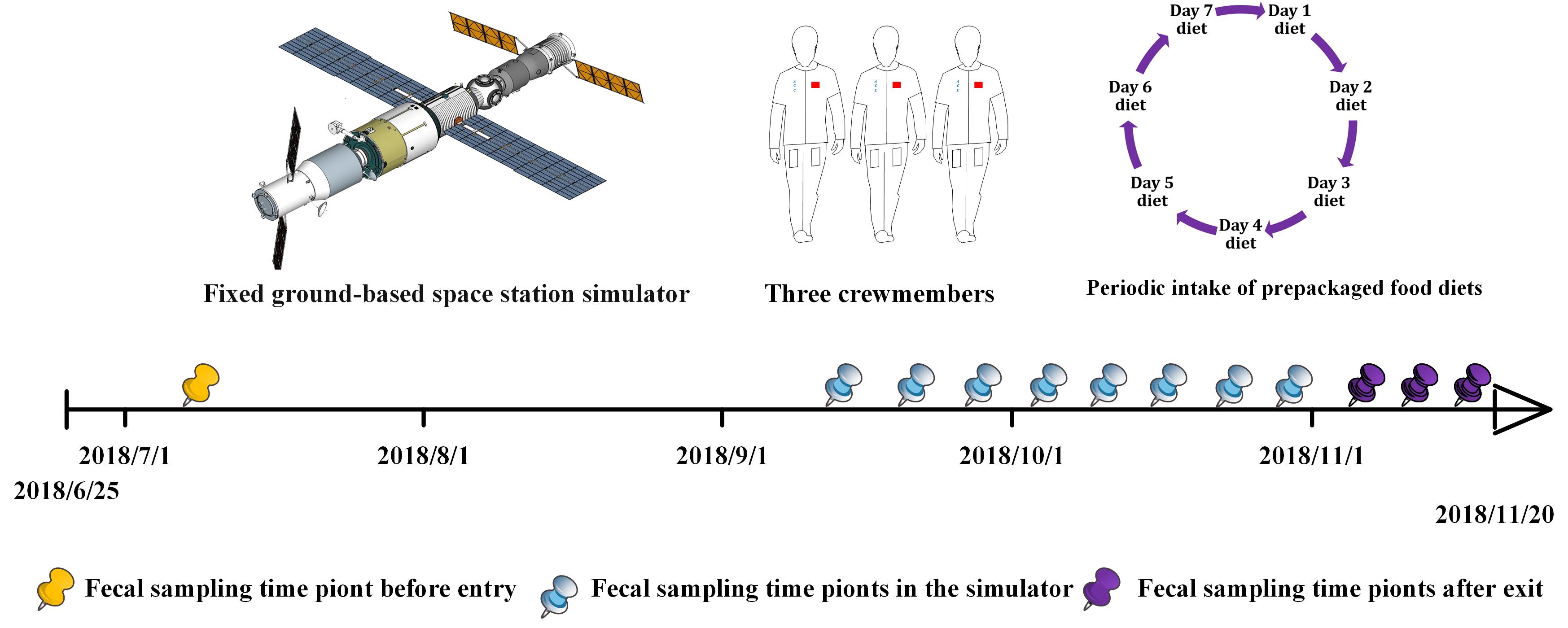

Supplement: Supplementary file 1 [file Image_1.jpg]

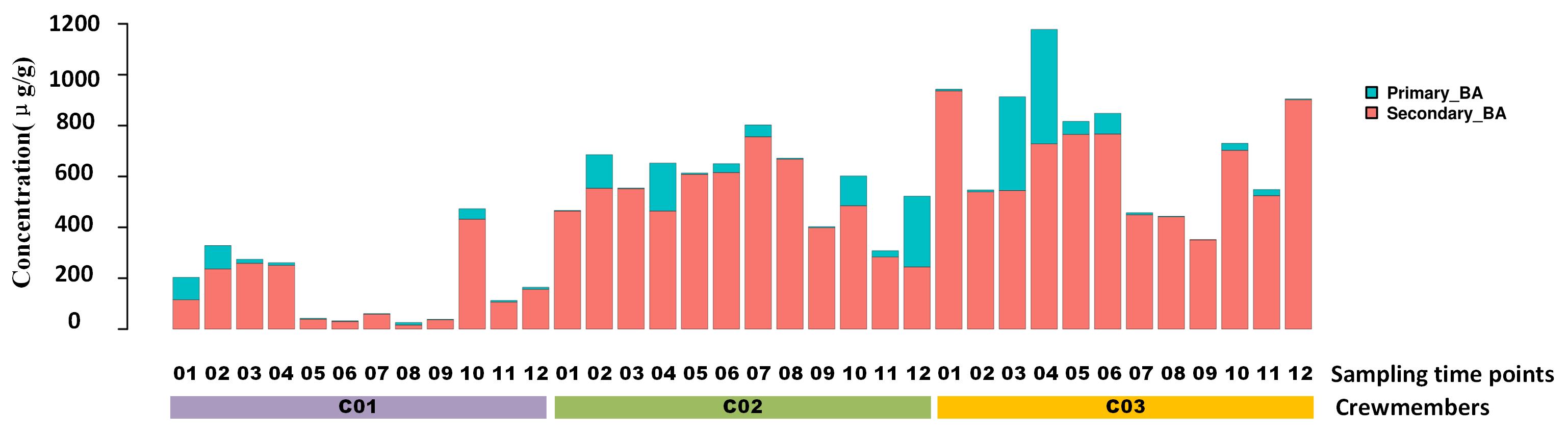

Supplement: Supplementary file 2 [file Image_2.jpg]

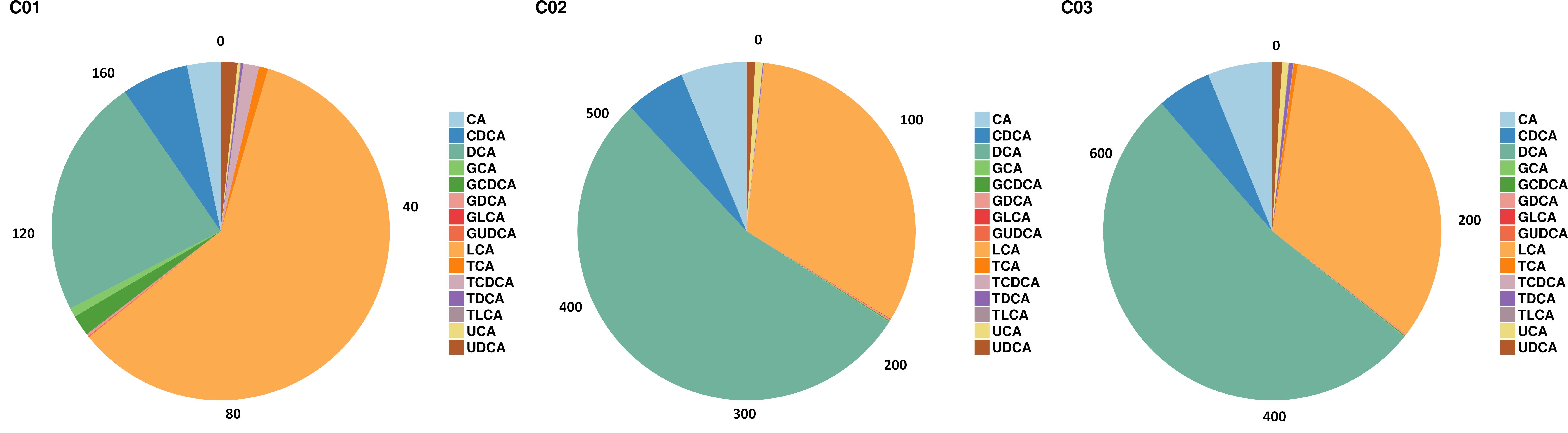

Supplement: Supplementary file 3 [file Image_3.jpg]

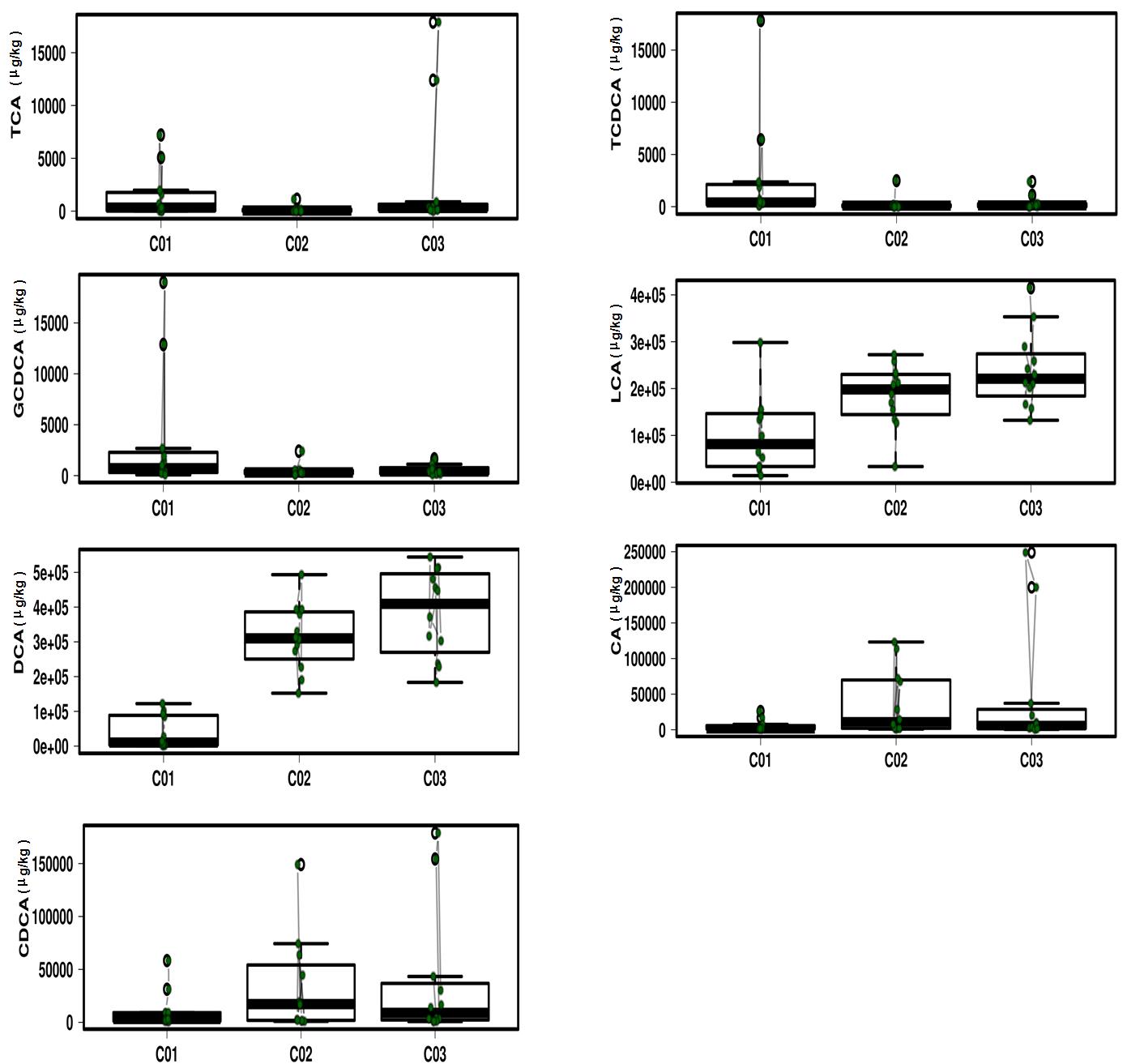

Supplement: Supplementary file 4 [file Image_4.jpg]

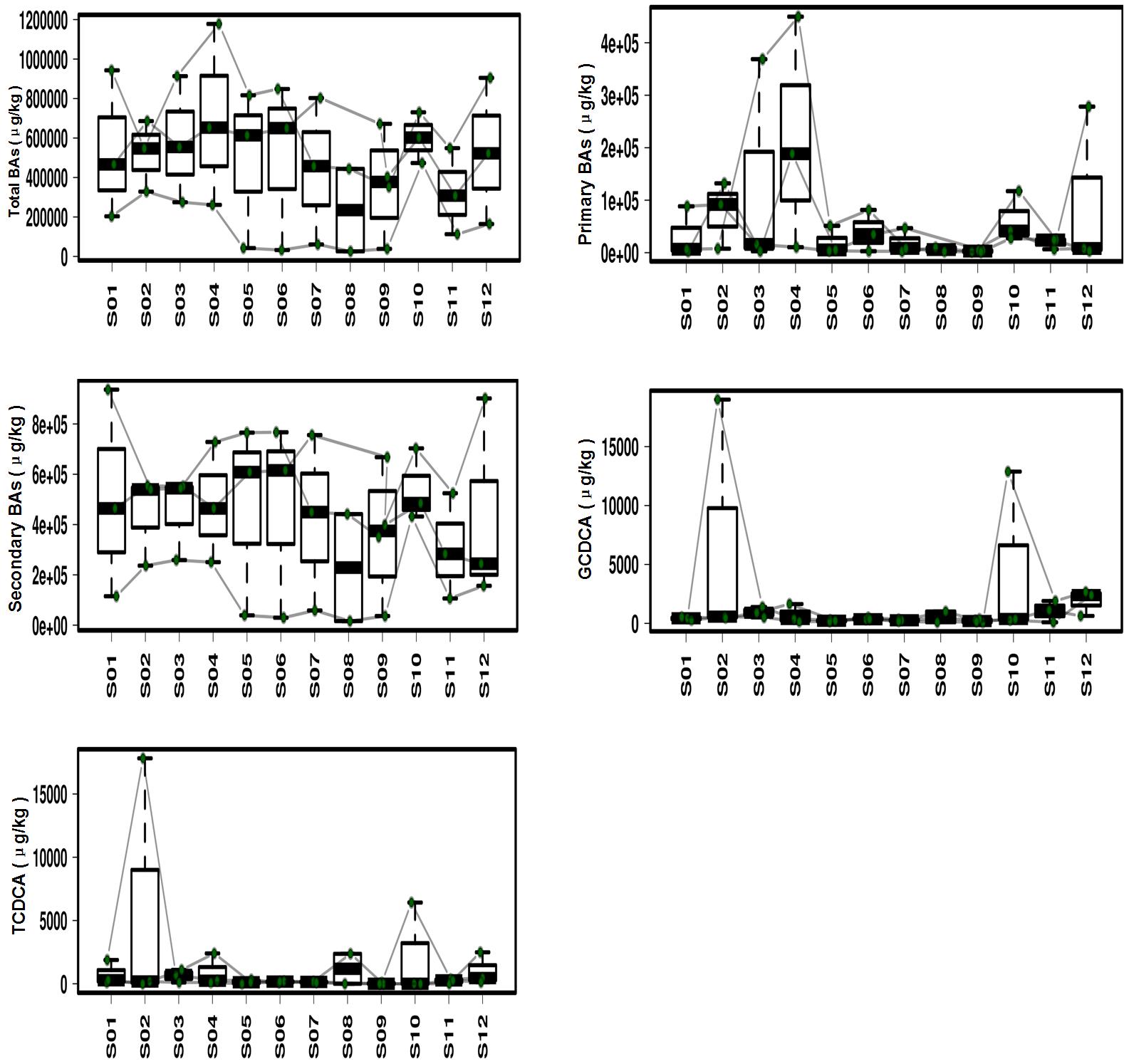

Supplement: Supplementary file 5 [file Image_5.jpg]

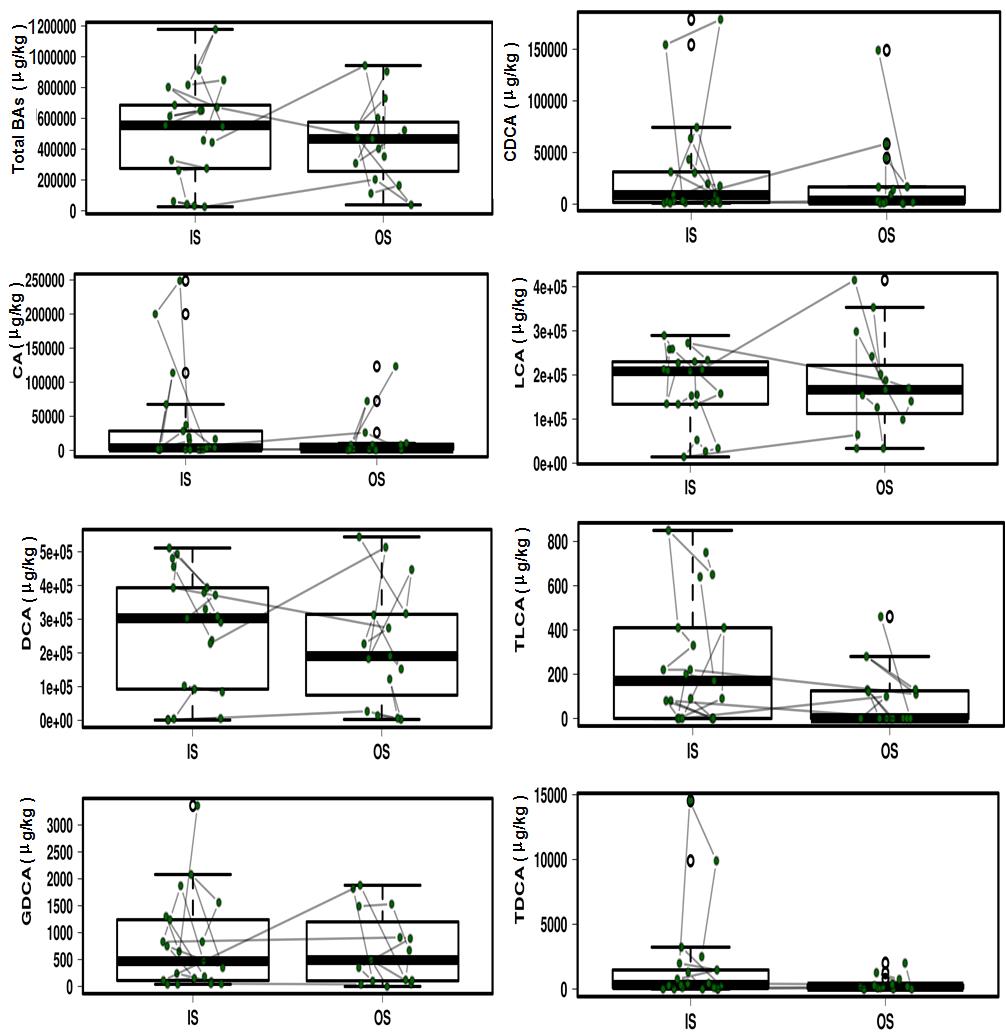

Supplement: Supplementary file 6 [file Image_6.jpg]
