# Supplementary material for: Fecal Bile Acids Profile of Crewmembers Consuming the Same Space Food in a Spacecraft Simulator
Source: Front Physiol. 2021 Oct 1;12:593226. doi: 10.3389/fphys.2021.593226 (PMC8517451; doi:10.3389/fphys.2021.593226)
Supplement: Supplementary file 7 [file Table_1.pdf]

Table S1 Major information of crewmembers and proportion of energy supply of the scheduled periodic diet

Crewmembers	Age	BMI (kg/m^2)[1]	Portion of energy supply (% kcal) (Mean ±SD)[2]		
			Protein	Fat	Carbohydrate
01	28	23.6 (23.0)	16.4±1.1	31.8±1.0	51.6±1.5
02	32	25.0 (24.3)	16.6±1.5	30.9±0.4	52.4±1.5
03	30	25.2 (24.6)	16.9±1.0	31.6±0.5	51.6±0.9

1 Body mass index (BMI) the day thirty days before entry and the extravehicular day (value in the brackets)
2 Average proportion of energy supply of the scheduled periodic diet
