# Supplementary material for: Fecal Bile Acids Profile of Crewmembers Consuming the Same Space Food in a Spacecraft Simulator
Source: Front Physiol. 2021 Oct 1;12:593226. doi: 10.3389/fphys.2021.593226 (PMC8517451; doi:10.3389/fphys.2021.593226)
Supplement: Supplementary file 8 [file Table_2.pdf]

Table S2 Summary of prepackaged food items of the scheduled 7 days periodic recipes

Meal	Food category	Day1	Day2	Day3	Day4	Day5	Day6	Day7
Breakfast	Staple food	Purple rice porridge	Millet porridge	Corn porridge	Buckwheat porridge	Rice porridge	Millet porridge	Corn porridge
	Ready to eat food	Coconut Bread	Sweet bread	Flowers sandwich bread	Baked Cake	Coconut bread	Sweet bread	Flower sandwich bread
	Non-staple food	Stewed Pleurotus eryngii	Capsicum potato	Stir-fried mushroom with minced meat	Spicy sea cabbage	Spicy Fennel peanut	Vegetarian fried Yam, fungus and celery	Stewed ballonflower
	Ready to eat food	Spiced egg	Spiced quail eggs	Spiced egg	Spiced quail eggs	Spiced egg	Spiced quail eggs	Spiced egg
		Pickled mustard tuber	Pickled turnip	Pickled mustard tubermustard tuber	Pickled turnip	Pickled mustard tuber	Pickled turnip	Pickled mustard tuber
	Condiment	Blueberry sauce	Ketchup	Blueberry sauce	Ketchup	Blueberry sauce	Ketchup	Blueberry sauce
Lunch	Staple food	Steamed rice	Stir-Fried Rice with Meat and Vegetables	Stir-Fried Rice with Soy Sauce	Steamed rice	Stir-Fried Rice with Meat and Vegetables	Stir-Fried Rice with Soy Sauce	Steamed rice
		Vegetarian rice-flour noodles	Sesame rice cake	Zongzi	Assorted rice	Vegetarian rice-flour noodles	Sesame rice cake	Zongzi
	Non-staple food	Hot and sour Bamboo shoot slice	Stir-Fried Pine nuts and corn	Spicy kelp	Oil stewed bamboo shoots	Sweet beans	Stir-Fried Lettuce and Pleurotus eryngii	Fresh spicy chayote
		Stir-Fried Black pepper beef strip	Braise potato and beef	Braised Pork chops	Stir-Fried Fungus with meat strip	Cooked Spicy lamb meat	Stir-Fried Cabbage with rabbit meat	Bitterned Donkey meat
	Rehydration vegetable soups	Tomato and egg soup	Seaweed and egg soup	Wild mushroom hibiscus soup	Fresh fragrant mushroom soup	Cabbage beef soup	Cabbage and tofu soup	Fresh vegetable chicken soup
	Condiment	Sweet chili sauce	Garlic sauce	Sweet chili sauce	Garlic sauce	Sweet chili sauce	Garlic sauce	Sweet chili sauce
Supper	Staple food	Fried noodles with Radish and ham	Fried noodles with Bamboo shoots and pork	Fried noodles with Radish and ham	Fried noodles with Bamboo shoots and pork	Fried noodles with Radish and ham	Fried noodles with Bamboo shoots and pork	Fried noodles with Radish and ham
		Green onion pancake	Rice cake	Rice cake	Salty cake	Green onion pancake	Steamed Pork Dumplings	Steamed Pork Dumplings
	Non-staple food	Fried water bamboo with Bacon	Stir-Fried Homemade tofu	Stir-Fried Eleocharis dulcis with tofu	Stir-Fried Vigna unguiculata with Minced meat	Stir-Fried Delicious kale	Stir-Fried Burdock with carrot	Honey stewed sun fruit
		Braised Bazhen chicken	Braised Milky chicken with corn kernels	Roast chicken leg	Stir-Fried Carrot with duck	Spicy duck meat	Spicy pigeon meat	Braised goose meat with Pepper
	Rehydration vegetable	Freeze-dried Shrimp with canola	Freeze-dried Chrysanthemum with Chicken oil	Freeze-dried Oyster sauce with lettuce	Freeze-dried salty spinach	Freeze-dried Sauced Lactuca sativa L.	Freeze-dried Chrysanthemum with Chicken oil	Freeze-dried Oyster sauce with lettuce
	Condiment	Spicy sauce	Seafood sauce	Spicy sauce	Seafood sauce	Spicy sauce	Seafood sauce	Spicy sauce
Snacks	Ready to eat food	Grain biscuit	Crisp biscuit	Compressed biscuits with Almond	Compressed biscuits with Cocoa	Grain biscuit	Crisp biscuit	Moon cake
		Stir-fried shrimp	Spicy pork meat	Stewed goose meat	Spicy tuna	Bittern Chicken gizzard	Spiced flounder	Spiced beef
		Canned apple	Canned pineapple	Canned fruits	Canned apple	Canned pineapple	Canned fruits	Canned apple
		Spiced dried bean	/	Spicy dried bean curd	/	Cheese	/	Milled Red bean and coix seed
		Peanut and almond	Chocolate	Peanut and almond	Chocolate	Peanut and almond	Chocolate	Peanut and almond
	Rehydration drink	Orange juice	Mango juice	Pineapple juice	Lemon juice	Peach juice	Apple juice	Black tea
