# Supplementary material for: Fecal Bile Acids Profile of Crewmembers Consuming the Same Space Food in a Spacecraft Simulator
Source: Front Physiol. 2021 Oct 1;12:593226. doi: 10.3389/fphys.2021.593226 (PMC8517451; doi:10.3389/fphys.2021.593226)
Supplement: Supplementary file 9 [file Table_3.pdf]

Table S3 List of the 16 kinds of bile acids investigated in this study

No.	BAs	Acronyms	Forms
1	Chenodeoxycholic acid	CDCA	Primary BA
2	Cholic acid	CA	Primary BA
3	Ursocholic acid	UCA	Secondary BA
4	Lithocholic acid	LCA	Secondary BA
5	Deoxycholic acid	DCA	Secondary BA
6	Ursodeoxycholic acid	UDCA	Secondary BA
7	Glycolithocholic acid	GLCA	Conjugated BA
8	Glycochenodeoxycholic acid	GCDCA	Conjugated BA
9	Tauroursodeoxycholic acid	TUDCA	Conjugated BA
10	Taurolithocholic acid	TLCA	Conjugated BA
11	Taurodeoxycholic acid	TDCA	Conjugated BA
12	Taurocholic acid	TCA	Conjugated BA
13	Taurochenodeoxycholic acid	TCDCA	Conjugated BA
14	Glycocholic acid	GCA	Conjugated BA
15	Glycoursodeoxycholic acid	GUDCA	Conjugated BA
16	Glycodeoxycholic acid	GDCA	Conjugated BA
